# Origin and Status of Homologous Proteins of Biomineralization (Biosilicification) in the Taxonomy of Phylogenetic Domains

**DOI:** 10.1155/2013/397278

**Published:** 2013-06-13

**Authors:** Igor E. Pamirsky, Kirill S. Golokhvast

**Affiliations:** ^1^Analytical Centre of the Mineralogical and Geochemical Researches, Institute of Geology and Nature Management FEB RAS, 1 Relochny Lane, Blagoveshchensk, Russia; ^2^Department of Oil and Gas Deal, Laboratory of Nanotoxicology, Far Eastern Federal University, 37 Pushkinskaya Street, Vladivostok, Russia

## Abstract

The taxonomic affiliation (in the systematisation of viruses, and biological domains) of known peptides and proteins of biomineralization (silicateins, silaffins, silacidins and silicase) and their primary structure homologues were analyzed (methods *in silico*; using Uniprot database). The total number of known peptides and proteins of biosilicification was counted. The data of the quantitative distribution of the detected homologues found in nature are presented. The similarity of the primary structures of silaffins, silacidins, silicateins, silicase, and their homologues was 21–94%, 45–98%, 39–50%, and 28–40%, respectively. These homologues are found in many organisms, from the Protista to the higher plants and animals, including humans, as well as in bacteria and extracellular agents, and they perform a variety of biological functions, such as biologically controlled mineralisation. The provisional classification of these biomineralization proteins is presented. The interrelation of the origin of the first organic polymers and biomineralization is discussed.

## 1. Introduction

Minerals formed with the participation of various organisms are diverse [1–3]. Such mineral formations are called biominerals, and the process of their formation is known as biomineralization. Biologically mediated and biologically controlled biomineralizations are distinct [4]. As a rule, the latter products involve biominerals of endogenous origin. At the same time, calling the formation of some endogenous biominerals, such as urinary stones, a controlled process is difficult. Considering the latest international research in this field [5–15], controlled biomineralization, in a broad sense, should be understood as not only a process of the formation of mineral particles but also their subsequent transformations, that is, metabolism. The metabolism of physiogenic biominerals, the biochemical processes of which are determined genetically and proceed with the direct participation of a number of molecules of protein origin, is of special interest.

Peptides and proteins involved biomineralization can be termed “proteins of biomineralization” (POB) and are divided into the following groups: (1) peptides and polypeptides, which form an organic matrix on which minerals are formed, (2) enzymes that catalyse the formation of inorganic structures, (3) enzymes that catalyse the hydrolysis of inorganic structures, and (4) proteins transporting the structural components of biominerals.  The high relevance of a study on POBs is caused by the depth of fundamentality, especially with respect to the interconnection of biology, geology, and medicine and the importance of the practical value, such as the synthesis of materials with specific functions for various technologies. However, many questions about the regularities and features of the biomineralization mechanisms (in particular, from the point of view of the POB) remain open, and the search for structural and functional homologues of known POBs in different organisms appears to represent one solution for these issues. In this regard, beginning the study of the most ancient primitive organisms, which were first on the earth, to enable the construction of a biomineral using endogenous protein molecules appears logical. The magnetotactic bacteria forming magnetite belong to these organisms as well as diatoms and sponges, forming silicon dioxide, and other organisms.

Today, representatives of biosilification proteins cover all groups of the above POB classifications. Prominent representatives of these proteins are silaffins and silacidins in diatoms and silicateins and silicase in river and sea sponges. Silaffins and silacidins (phosphoproteins) are small peptides that catalyse the formation of silica nanospheres from silicic acid and control their size, playing a central role in the formation of the cell walls of diatoms. Silicateins catalyse the generation of amorphous silica from silicic acid esters, participating in the formation of the silicon skeleton. Silicase is the only known enzyme that performs the depolymerisation of silica. Silicon transporters (SIT) are proteins involved in the transmembrane transport of silicic acid. This paper is devoted to the study of enumerated peptides and proteins (silicon transporter (SIT) data not shown).

## 2. Materials and Methods

The amino acid sequences of all the studied proteins, except silicase and silacidins, were taken from the computational biology database server, UniProt (release 2012_10, http://www.uniprot.org). The data on silicase were taken from the paper by Schröder et al. [14], and the data on silacidins were taken from the paper by Richthammer et al. [15]. A comparative study of the homology of the amino acid sequences of peptides and proteins was performed using the same server (at the time of data retrieval from the database, there was information on approximately 30 million sequences). For comparison of the primary structures of biomineralization proteins and their families and groups, the multiple sequence alignment mode “Align” was used, implemented through “ClustalW 2.0.12” (with the mode settings not configured). The search for protein homologues was performed by pairwise alignment with the tool “BLAST” (BLASTP 2.2.26, Sep-21-2011, implemented through NCBI), as provided by the server, with the following parameters: database - UniProtKB, Threshold - 0, 1-0,0001, Matrix - Auto, Filtering - None (for proteins)/Filter low complexity regions (for peptides), Gapped - yes, Hits - 250. A similar approach was used in other studies [16–18].

Homologues of the primary structure were examined for three typical representatives of each group of proteins and peptides (except silicase). For the silaffin predecessor, short silaffins were chosen from *Cylindrotheca fusiformis* (ID Q9SE35, 265 Am), and silaffins were chosen from *Thalassiosira pseudonana* (ID Q5Y2C0, 231 Am; ID Q5Y2C1, 485 Am). For silacidins, natsilacidin A and silacidins B and C were selected. For silicateins, silicatein-*α* (ID B1GSK9, 334 Am) was selected from *Geodia cydonium*, and silicateins A1 (ID B5B2Z1, 329 Am) and A2 (ID B5LT52, 329 Am) were selected from *Latrunculia oparinae*.

## 3. Results and Discussion 

### 3.1. The Distribution of the Homologues with respect to Origin and Their Taxonomic Affiliation

**Figure 1:**
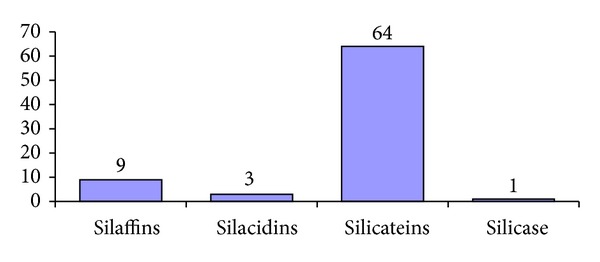
The number of known proteins of biomineralization in diatoms and sponges (from UniProt and the literature by the end of 2012).

The number of studied biomineralization peptides and proteins, with the information detailed in the literature and submitted to the Uniprot database (with the polypeptide chains of some proteins represented partially in the database because their amino acid sequences have not been fully deciphered), is shown in Figure 1. Interestingly, since the discovery of silicase, none of its counterparts have been found.

The quantitative distribution of homologues with respect to origin and their taxonomic affiliations in the systematisation of viruses and biological domains are presented in Tables 1 and 2. Due to the peculiarity of the method used, the obtained data do not imply an affiliation of other (unknown, undiscovered, or not included in the number of results due to the limited number of issued results) and only show homologues to the taxa mentioned in the Tables.

### 3.2. Matrix Proteins (Silaffins and Silacidins)

#### 3.2.1. Silaffins

The silaffins (four individual polypeptides and a predecessor of short silaffins) presented in the database belong to two species of diatoms. Homology was found only between polypeptides from *T. pseudonana*, which were Q5Y2C1 and Q5Y2C2 (485 and 501 Am) and Q5Y2C0 and B8BRK6 (include to 231 Am), and was 99% for each pair (names of the proteins are not shown, but the identification numbers are in the database). The short silaffins 1B, 1A2, and 1A1 (peptide lengths of 29, 18, and 29 Am from *C. fusiformis*) are identical to each other up to 32–60%, but their common polypeptide predecessor (265 Am) from *T. pseudonana* is not homologous.

The proteins identical to the studied silaffins up to 21–94% (matrix blosum 62; *E* value from 0 to 5.0 × 10^−6^) are found mainly in cellular organisms (eukaryotes and bacteria) and in some viruses (Tables 1 and 2). The biological and molecular functions of the majority of found homologues are not associated with biomineralization or are unknown. Biomineralization proteins are detected only among silaffin-1 homologues. There are approximately 80 dentin sialophosphoprotein (DSPP): noncollagenous matrix dentin proteins in mammals regulating the mineralisation and the size and shape of apatite crystals. The presence of phosphorylated amino acids in these proteins is an additional similarity to silaffins. The majority of serines in silaffins are known to be phosphorylated and are a source of anions in the formation of silica [11]. Lustrin A from the mollusc *Haliotis rufescens* is another homologue of silaffin-1, reinforcing the pearl layer of shells and pearls. Previously, Shen et al. [5] noted the similarity of the structures of lustrin, proteins (frustulins) forming the silicon skeleton of diatoms, extracellular matrix proteins composing the mineralised matrix of bone and dental tissues in mammals, and proteins composing avian egg shells. Some proteins (B8LDT2, B8LDT6, and B3ITC3) of the cellular wall of the diatom* T. pseudonana* and the bivalve *Crassostrea nippona*, with its calcite shell, are likely shown in the list of homologues but are uncharacterised and thus may participate in biomineralization.

Cementing or bonding proteins are worth paying attention to along with the other silaffin-1 homologues, such as the silk proteins of the silkworm *Bombyx mori*, the lacewing *Mallada signata*, the emby *Aposthonia gurneyi*, and *Haploembia solieri*, which are used for building cocoons and spider passages, and the cementing protein 3B of the worm *Phragmatopoma californica* required for the binding of sand and sea shell remains in the construction of habitation. These proteins are also characterised by a generous amount of serine and repeating regions, but they are not related to biomineralization. However, a detailed comparative study of their expressed adhesion properties to different surfaces (including minerals) may help improve the understanding of the mechanism of matrix biomineralization protein action.

Mucins should be mentioned (silaffin Q5Y2C2 homologues), as presented by several dozen representatives (not involved in biomineralization). Some mucin-like proteins are known to participate in the process of the mineralisation of mollusc shells [22] as well as of the bone, teeth, and cartilage of vertebrates [23]; that is, their biological functions are similar to those of silaffins. However, there were no mucin-like mineralising proteins in the list of the 250 homologues. A separate comparison of a typical example of such proteins (shellfish protein Q9BKM3; selected randomly) with Q5Y2C2 showed a low degree of sequence similarity of approximately 17%, which explains the results of the search for homologues in the database.

#### 3.2.2. Silacidins

**Figure 2:**
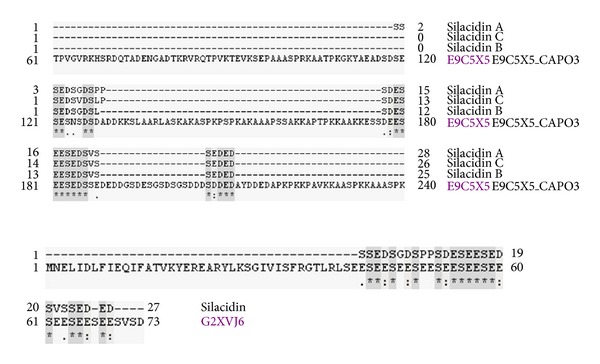
Example of an alignment of the amino acid sequences of silacidins A (silacidin A, silacidin), B (silacidin V), C (silacidin S), DNA topoisomerase I *Capsaspora owczarzaki* (E9C5X5), and a undocumented protein from the fungus *Botrytis cinerea* (G2XVJ6).

Homology between the A, B, and C silacidins was approximately 86%. A homology search revealed 54, 17, and 14 results for the A, B, and C silacidins, respectively. The identity to the detected proteins is 45–98% (with matrix pam 30). A significant difference in the length (2,7–64 times) between the silacidin polypeptide chains and these proteins explains the low similarity at the level of entire sequences (*E* value of 2.0 × 10^−3^ to 6.7 × 10^−2^). At the same time, there is high homology with the individual chains sections of most found proteins (an example is shown in Figure 2). The functions of the majority of these homologues are currently unknown, and the remainder of the proteins mainly contain zinc ions and nucleic acids. In the mode of the high statistical threshold of significance (value ≥ 10) in the list of homologues, biomineralization proteins were reflected, such as the dentin matrix protein from the proboscis dog *Rhynchocyon petersi* and lemur *Lemur catta* and osteopontin-bone proteins from mouse *Mus musculus* and sea carp *Sparus aurata*.

Asterisks indicate identical amino acids, and “·” and “:” indicate chemically similar amino acids. The numbers indicate the ranges of the amino acid residues corresponding to the line. The topoisomerase sequence is incomplete.

### 3.3. Silica Polymerising (Silicateins) and Depolymerising (Silicase) Enzymes

#### 3.3.1. Silicateins

With the method used, the degree of the identity of the primary structure of silicateins was approximately 40–99%. There was no one protein with a serine catalytic centre among all known silicatein homologues (homology of 39–50% with a matrix blosum of 62 and *E* value of 3.0 × 10^−89^ to 1.0 × 10^−88^). Cathepsins L, S, and K (with the cysteine type catalytic centre) play the primary role in a wide range of unicellular and multicellular eukaryotes (all classes are listed in Table 2). If cathepsins L and S are not related to biomineralization processes, cathepsin K (tissue-specific enzymes of osteoclasts that break down bone matrix proteins) is only indirectly related to biomineralization (the direct contact with the formation of enzyme-substrate complexes is unknown). The molecular and biological functions of the other homologues are unknown.

#### 3.3.2. Silicase

The silicase homology search of the sponge *S. domuncula* showed that all the proteins that are identical to it at 28–40% (matrix blosum 62, *E* value of 8.0 × 10^−43^ to 1.0 × 10^−17^) are related to carbonate dehydratase. These homologues are predominantly found in organisms from the “Cambrian explosion” (see Tables 1 and 2), and silicase itself is most likely the “youngest” enzyme of all POBs. The biological roles of the homologues are known to varying degrees. For example, some are involved in bone resorption and osteoclast differentiation, but considering the mechanism of action, the analogy with silicase is impossible to draw.

Despite the relatively high level of similarity (and in most cases, conservation) in the primary organisation, the immediate analogues among the found silicase silicatein homologues are not shown. In the case of silicateins (serine protease), these omissions are explained by the difference in the structure of the catalytic centre from cysteine cathepsins. Other serine proteases may be able to operate with silicic acids. Silicase, as a representative of carbonic anhydrase, is of great interest to us. We also did not find information about the direct functional analogues of sponge silicase in the literature.

The silicase enzymes are supposed to be present in the silicate bacteria responsible for the destruction of Si–O bonds in the crystal lattice of clay minerals and the Si–C bonds in organosilicon compounds, but these enzymes are not isolated in a pure form [24, 25]. Some cellular organisms may produce silicase-like enzymes under certain circumstances. Such enzymes may well contribute to the assimilation (exchange) of silicon, entering the organism with water and nutriments. It is impossible not to take into account the existence of the lithophagy phenomenon in mammals [26]. Various silicon clays are widely used in modern medicine, including orally. Adhering to certain logic, it makes sense to use representative examples, such as the human enzyme chitotriosidase from the family of chitinases that implements the hydrolysis of chitin, which is a characteristic of the covering tissue of fungi, insects, and crustaceans. Although this substrate of chitinase is not a structural element of the human body, specific cells produce these enzymes under certain situations (in the case of some mucopolysaccharidoses) [27].

## 4. The Origin of Biomineralization Proteins

Silaffin homologues are found amongst representatives of all the kingdoms of organisms and virus taxa (see Table 2). A similar pattern is observed for silacidin homologues. Thus, it seems logical that matrix proteins (not even involved in biomineralization) combined with other plastic organic molecules form the basis of subcellular structures and cells in general. The POB homologues and proteins of biomineralization themselves detected in the representatives of specified taxa may have common ancestors, but such a hypothesis requires a detailed phylogenetic analysis. However, from the point of view of evolution, bacteria and viruses are of the greatest interest to us. Geological findings indicate that bacteria are the most ancient organisms on the earth. At the same time, the one-time (random) emergence of complex living systems such as bacteria is unlikely, but modern science is yet unable to answer clearly what transitional forms (stages) preceded the emergence of single-celled organisms. If subcellular agents (viruses) preceded a cellular form of life, they had to be carriers of the first matrix proteins. However, virus fossils still have not been found. In any case, initially for the construction of organic biological systems in the same time period, a set of organic molecules had to be specific, including those able to fulfil structural and metabolic functions. Where did these organic molecules for the construction of such systems, especially those proteins for which their synthesis was stipulated genetically, come from? According to some hypotheses [3, 28–33], minerals, acting as templates, catalysts, and/or metabolites, promoted the synthesis and interaction of organic molecules and the emergence of life. In an article devoted to mineral evolution, Hazen et al. [3] highlighted the probability that matrix (structural) proteins should be among the first organic polymers and that the emergence of life is associated with the achievement of a minimum level of mineral evolution. Kostetsky [31, 32] provided a fairly detailed and universal scenario of the simultaneous abiotic synthesis of nucleic acids and proteins (collagen, histones, and others) on apatite matrix (also on carbonate apatite, calcite, aragonite, cristobalite, and mica) and then through the formation of organic-crystalline complex, the emergence of protocells, which were derived from minerals, and the subsequent reproduction of the matrix mechanism, genetic code, DNA structure, and other crystal-chemical features. It follows that an inorganic matrix and its synthesis on organic molecules, including polymers, must have occurred at approximately the same time, which is hard to believe in practice. Nevertheless, the previous prebiotic synthesis of molecules does not exclude the emergence of unrelated protocells but does include homologous polypeptides and polynucleotides. This picture fits into Zavarzin's opinion [34], who hypothesised the existence of a “universal ancestor” to be logically contradictory, emphasising the obligatoriness of the diversity and functional complementarity of the original group of microorganisms. Presumably, the main metabolism types were formed no later than 3.5 billion years ago (cyanobacteria, as discussed), and replacing inaccessible metals with those available in the enzyme structure was likely one of the main methods of early metabolic evolution [28]. Bacteria copied all the possible abiotic reactions associated with clay minerals, with the main difference being their speed [35]. Thus, biomineralization is essentially the reverse process, with biological molecules and supramolecular structures acting as intermediaries of mineralisation [36].

The repeated occurrence of biomineralization in the process of evolution as part of metabolism, depending on the environmental conditions, may mean that organisms already had the necessary “tools” (proteins). Thus, proteins able to “work” with biominerals (especially matrix proteins) may have been found before, and generic ancestors of these organisms may have already had a minimal set of such proteins. In this regard, the mass mineralisation in the Cambrian and further diversification of mineral skeletons in the Ordovician (accompanied by significant changes in the environment) are logical outcomes when many new taxa were “ready” to metabolise the compounds of calcium, phosphate, and silicon.

## 5. Conclusions

The proteins with primary structures that are moderately and highly homologous to silaffins, silacidins, silicase, and silicateins occur in many different organisms, from Protozoans to the higher plants and animals, including humans, as well as in bacteria and extracellular agents.

The biological and molecular functions of these homologues vary (e.g., protein binding, binding of metal ions, transferase activity, and proteolysis), but most of them are not directly related to the formation of biomineral particles. Only a few homologues are direct analogues of silaffins and silacidins or are able to participate only indirectly in biomineralization (silicase silicatein homologues).

The data on silaffins and silacidins allow the evolutionary relationships of different biomineralization types to be considered more closely. The formation mechanisms of silica, phosphate, and calcium carbonate particles on such protein matrices are likely fundamentally similar.

Research in this area enhances the understanding of the mechanisms of the formation of physiogenic and pathogenic biominerals as well as the origins of life and the coevolution of the living and nonliving.

## Figures and Tables

**Table 1:** Quantitative distribution of the homologues with respect to origin.

Domains of biological taxonomy	Titles of POB			
	Silaffins	Silacidins	Silicateins	Silicase
Viruses	1%	0.8%	—	—
Archaea	0.6%	—	—	—
Bacteria	38.4%	—	—	—
Eukaryote	60%	99.2%	100%	100%
Total number of homologues	741	82	686	249

Note: peptides and proteins belonging to the group of proteins under study shown in the list of homologues were not considered.

**Table 2:** Taxonomy homologues proteins of biomineralization.

Taxonomy of viruses and organisms	Age (billions of years)	POB			
		1	2	3	4
Mimiviridae, Herpesviridae, Baculoviridae and so forth (vira**)	Fossils are not found [19]	+	+		
Bacilli, Gammaproteobacteria and so forth (bacteria)	3.8–3.5* [19]	+			
Dictyosteliomycota	—	+	+	+	
Lobosea	0.75 [20]			+	
Heterolobosea	—	+			
Kinetoplastida	0.099–0.093 [21]		+		
Conoidasida	—	+			
Aconoidasida	—	+			
Oligohymenophorea	—	+			
Demospongiae	0.542–0.516 [21]			+	+
Tricoplacia	—	+	+	+	
Hydrozoa	0.635–0.542 [21]	+		+	
Anthozoa	0.635–0.542 [21]			+	
Holothuroidea	0.513–0.505 [21]			+	
Chromadorea	—	+	+	+	+
Bdelloidea	—			+	
Branchiopoda	0.520–0.516 [21]	+	+	+	
Monogenea	—			+	
Polychaeta	0.520–0.516 [21]			+	
Gastropoda, Bivalvia	0.542–0.252 [21]	+		+	
Arachnida	0.418–0.416 [21]			+	
Insecta	0.412–0.391 [21]	+		+	+
Malacostraca	0.530–0.513 [21]			+	
Maxillopoda	0.268–0.265 [21]			+	+
Appendicularia	—		+	+	+
Leptocardii	0.541–0.485 [21]			+	
Actinopterygii	0.478–0.468 [21]	+		+	+
Amphibia	0.488–0.443 [21]	+		+	+
Aves	0.252–0.201 [21]	+		+	+
Mammalia	0.235–0.221 [21]	+	+	+	+
Homo sapiens***	0.005 [21]	+		+	+
Eurotiomycetes, Homobasidiomycetes and so forth (fungi)	0.048–0.46 [21]	+	+		
Phaeophyceae, Mamiellophyceae and so forth	0.485–0.150 [21]	+	+		
Liliopsida	0.122–0.112 [21]	+		+	
Magnoliopsida	0.388–0.383 [21]	+			

Note: 1: silaffins; 2: silacidins; 3: silicateins; 4: silicase; +: homologue present; **(classification ICTV); ***(human is presented as a species as an exception). *3.5 authentic finding in siliceous rocks, 3.8 problematic.
